# Human Cardiac ^31^P-MR Spectroscopy at 3 Tesla Cannot Detect Failing Myocardial Energy Homeostasis during Exercise

**DOI:** 10.3389/fphys.2017.00939

**Published:** 2017-11-27

**Authors:** Adrianus J. Bakermans, Jason N. Bazil, Aart J. Nederveen, Gustav J. Strijkers, S. Matthijs Boekholdt, Daniel A. Beard, Jeroen A. L. Jeneson

**Affiliations:** ^1^Department of Radiology, Academic Medical Center, University of Amsterdam, Amsterdam, Netherlands; ^2^Department of Physiology, Michigan State University, East Lansing, MI, United States; ^3^Biomedical Engineering and Physics, Academic Medical Center, University of Amsterdam, Amsterdam, Netherlands; ^4^Department of Cardiology, Academic Medical Center, University of Amsterdam, Amsterdam, Netherlands; ^5^Department of Molecular and Integrative Physiology, University of Michigan, Ann Arbor, MI, United States; ^6^Neuroimaging Center, Department of Neuroscience, University Medical Center Groningen, University of Groningen, Groningen, Netherlands

**Keywords:** myocardial energy metabolism, phosphorus-31 magnetic resonance spectroscopy, computational modeling, cardiac exercise stress testing, hypertrophic cardiomyopathy, energy homeostasis, ^31^P-MRS

## Abstract

Phosphorus-31 magnetic resonance spectroscopy (^31^P-MRS) is a unique non-invasive imaging modality for probing *in vivo* high-energy phosphate metabolism in the human heart. We investigated whether current ^31^P-MRS methodology would allow for clinical applications to detect exercise-induced changes in (patho-)physiological myocardial energy metabolism. Hereto, measurement variability and repeatability of three commonly used localized ^31^P-MRS methods [3D image-selected *in vivo* spectroscopy (ISIS) and 1D ISIS with 1D chemical shift imaging (CSI) oriented either perpendicular or parallel to the surface coil] to quantify the myocardial phosphocreatine (PCr) to adenosine triphosphate (ATP) ratio in healthy humans (*n* = 8) at rest were determined on a clinical 3 Tesla MR system. Numerical simulations of myocardial energy homeostasis in response to increased cardiac work rates were performed using a biophysical model of myocardial oxidative metabolism. Hypertrophic cardiomyopathy was modeled by either inefficient sarcomere ATP utilization or decreased mitochondrial ATP synthesis. The effect of creatine depletion on myocardial energy homeostasis was explored for both conditions. The mean *in vivo* myocardial PCr/ATP ratio measured with 3D ISIS was 1.57 ± 0.17 with a large repeatability coefficient of 40.4%. For 1D CSI in a 1D ISIS-selected slice perpendicular to the surface coil, the PCr/ATP ratio was 2.78 ± 0.50 (repeatability 42.5%). With 1D CSI in a 1D ISIS-selected slice parallel to the surface coil, the PCr/ATP ratio was 1.70 ± 0.56 (repeatability 43.7%). The model predicted a PCr/ATP ratio reduction of only 10% at the maximal cardiac work rate in normal myocardium. Hypertrophic cardiomyopathy led to lower PCr/ATP ratios for high cardiac work rates, which was exacerbated by creatine depletion. Simulations illustrated that when conducting cardiac ^31^P-MRS exercise stress testing with large measurement error margins, results obtained under pathophysiologic conditions may still lie well within the 95% confidence interval of normal myocardial PCr/ATP dynamics. Current measurement precision of localized ^31^P-MRS for quantification of the myocardial PCr/ATP ratio precludes the detection of the changes predicted by computational modeling. This hampers clinical employment of ^31^P-MRS for diagnostic testing and risk stratification, and warrants developments in cardiac ^31^P-MRS exercise stress testing methodology.

## Introduction

The human heart requires a continuous and adequate supply of energy to guarantee myocardial contractility that is required to support blood circulation. Myocardial energy homeostasis is maintained primarily by oxidative phosphorylation of adenosine diphosphate (ADP) in cardiomyocyte mitochondria. A disruption of myocardial energy homeostasis may impair mechanical function of the heart (Tewari et al., 2016b). Indeed, impaired mitochondrial function can lead to a life-threatening state of heart failure (Neubauer, 2007; Brown et al., 2016). Therefore, homeostasis of myocardial energy metabolism and its (mal-)adaptation in heart disease has been an important area of cardiovascular research (Taegtmeyer et al., 2016).

Phosphorus-31 MRS (^31^P-MRS) is a non-invasive and non-ionizing imaging modality that is uniquely capable of probing *in vivo* myocardial high-energy phosphate metabolism. This technique can quantify the steady-state myocardial phosphocreatine (PCr) over ATP concentration ratio (Bottomley, 2007), which has been commonly used to characterize the *in vivo* myocardial energy status. The PCr/ATP ratio is assumed to correlate with the cytosolic Gibbs free energy of ATP hydrolysis (ΔG_P_), the energy available to cardiomyocytes to do work. However, this assumption is valid, if and only if, the myocardial creatine content is either known or can be assumed to be unchanged compared with healthy hearts (Wu et al., 2009). It has long been known that the myocardial creatine content can be reduced in the diseased heart (Cowan, 1934; Herrmann and Decherd, 1939), thus complicating a straightforward interpretation of measured PCr/ATP ratios in patients. Furthermore, the measured myocardial PCr/ATP ratio only reports on the balance between ATP turnover rate and ATP synthesis at a specific steady-state. The underlying cause of any observed difference between the PCr/ATP ratio in heart disease and in the healthy heart cannot be identified without additional measurements. Indeed, measurements of the myocardial PCr/ATP ratio at multiple steady-states or during transition between steady-states of cardiac work may unmask underlying energy deficits in heart disease (Dass et al., 2015). Furthermore, such measurements would allow for a meaningful characterization of the (patho-)physiology of *in vivo* myocardial energy homeostasis guided by computational modeling and simulations of cardiomyocyte energy homeostasis (Balaban, 2006; Beard and Kushmerick, 2009), which ultimately may facilitate diagnosis and risk stratification in patients.

**Figure 1:**
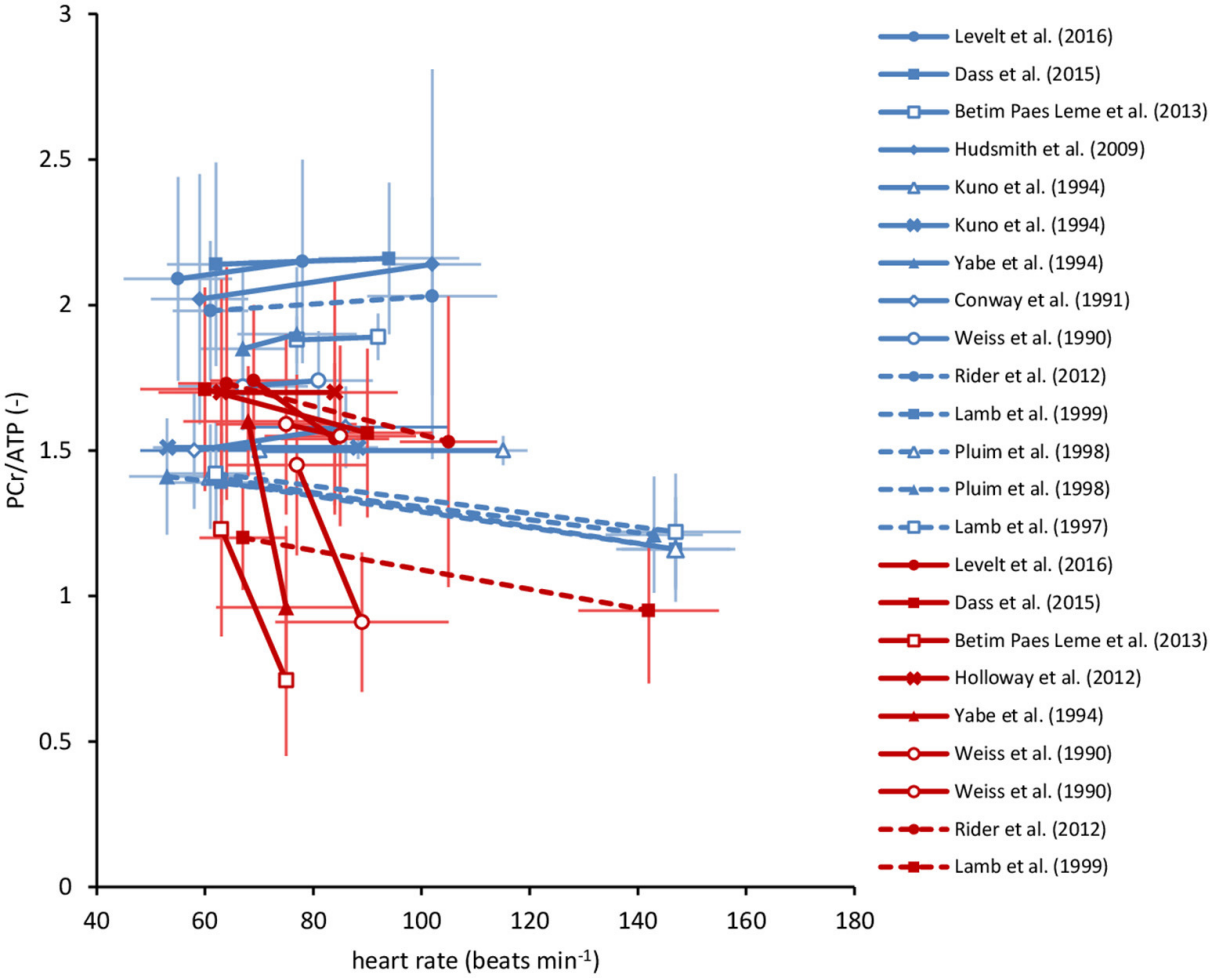
Overview of myocardial PCr/ATP ratios in human cardiac ^31^P-MRS stress testing reported in the literature. These studies involved measurements in healthy volunteers (blue) and various patient populations (red), at rest and during exercise (solid lines), or pharmacologically induced stress (dashed lines). Studies that documented both heart rate as well as the myocardial PCr/ATP ratio at rest and at increased cardiac work rates are included. Error bars represent *SD*. Note the large variability in the reported PCr/ATP ratios, both within as well as between studies. See literature for individual study details (Weiss et al., 1990; Conway et al., 1991; Kuno et al., 1994; Yabe et al., 1994; Lamb et al., 1997, 1999; Pluim et al., 1998; Hudsmith et al., 2009; Holloway et al., 2012; Rider et al., 2012; Betim Paes Leme et al., 2013; Dass et al., 2015; Levelt et al., 2016). ATP, adenosine triphosphate; PCr, phosphocreatine.

Obtaining reliable *dynamic* PCr/ATP ratios from the human heart during transitions between cardiac work rates is unrealistic (van Beek et al., 1998). Instead, ^31^P-MRS measurements of the myocardial PCr/ATP ratio at *multiple steady-states* of cardiac work rates are feasible. Pioneered almost three decades ago (Conway et al., 1988, 1991; Weiss et al., 1990; Kuno et al., 1994), ^31^P-MRS measurements of the *in vivo* human heart during exercise have recently regained interest (Hudsmith et al., 2009; Betim Paes Leme et al., 2013; Dass et al., 2015; Levelt et al., 2016). These studies typically consisted of steady-state ^31^P-MRS data acquisition at rest and at one additional steady-state of low-intensity exercise (heart rates of 60–100 beats min^−1^) or pharmacologically induced stress (Figure 1). Multiple measurements over a broader physiological range of cardiac work rates would be of major benefit for characterizing myocardial energy homeostasis. Indeed, successful implementation of more strenuous exercise regimens in clinical cardiac ^1^H-MRI protocols has recently been reported (La Gerche et al., 2013; Pflugi et al., 2015; Roberts et al., 2015; Barber et al., 2016), with maximal heart rates during supine in-magnet bicycle exercise exceeding 160 beats min^−1^ (La Gerche et al., 2013). However, ^31^P-MRS suffers from low sensitivity and poor measurement repeatability compared to ^1^H-MRI, compromising a quantitative evaluation of potentially subtle changes in myocardial energy homeostasis.

Here, we investigated whether the current standard of ^31^P-MRS methodology to measure *in vivo* myocardial PCr/ATP ratios typically implemented on clinical research MR systems is sufficient to discriminate between exercise-induced changes in steady-state myocardial energy metabolism in health and disease. Hereto, we determined and compared the precision in terms of measurement variability and repeatability of commonly used localized ^31^P-MRS methods to quantify the myocardial PCr/ATP ratio in healthy subjects at rest. To compare the precision of cardiac ^31^P-MRS measurements with another *in vivo* application of ^31^P-MRS, we also determined the precision of ^31^P-MRS measurements of the PCr/ATP ratio in stationary calf muscle. The results were then used in computational model simulations of the healthy heart and of hypertrophic heart disease, to estimate the magnitude of change that may be expected for the myocardial PCr/ATP ratio over a broad physiological range of cardiac work rates. Our findings show that improvements of the ^31^P-MRS measurement precision combined with in-magnet exercise at high intensities will be required for such investigations to become of diagnostic merit.

## Materials and methods

### Ethical approval

This study in healthy volunteers was carried out in accordance with the recommendations of the local institutional review board (Academic Medical Center, University of Amsterdam, Amsterdam, Netherlands) with written informed consent from all subjects. All subjects gave written informed consent in accordance with the Declaration of Helsinki prior to the MR examinations. The protocol was approved by the local institutional review board.

### *In vivo* ^31^P-MRS of the human heart

Eight volunteers (seven males and one female; age 32.4 ± 8.6 years; body mass index 23.5 ± 2.5 kg m^−2^) participated in this study.

All MR data were acquired on a 3 Tesla Philips Ingenia MR system (Philips Healthcare, Best, The Netherlands), equipped with a standard vendor-supplied ^31^P MR surface coil (140 mm; 51.8 MHz; Philips Healthcare) for radiofrequency transmission and signal reception. Heart rate was recorded and used to synchronize MR acquisitions via R-wave detection in the ECG signal. Subjects were positioned supine with the ^31^P MR surface coil carefully positioned on the chest covering the heart. Correct positioning of the coil was verified on ^1^H-MR scout images using a fiducial marker affixed to the coil center. Non-localized pulse-acquire ^31^P-MR spectra were obtained to assess metabolite T_1_ relaxation time constants using conventional saturation recovery experiments: repetition time (TR) 1,000–1,500–2,000–3,000–4,000–6,000–8,000–10,000 s, 4 averages/TR, γ-ATP on-resonance, 2,048 acquisition points, bandwidth 58 ppm.

**Figure 2:**
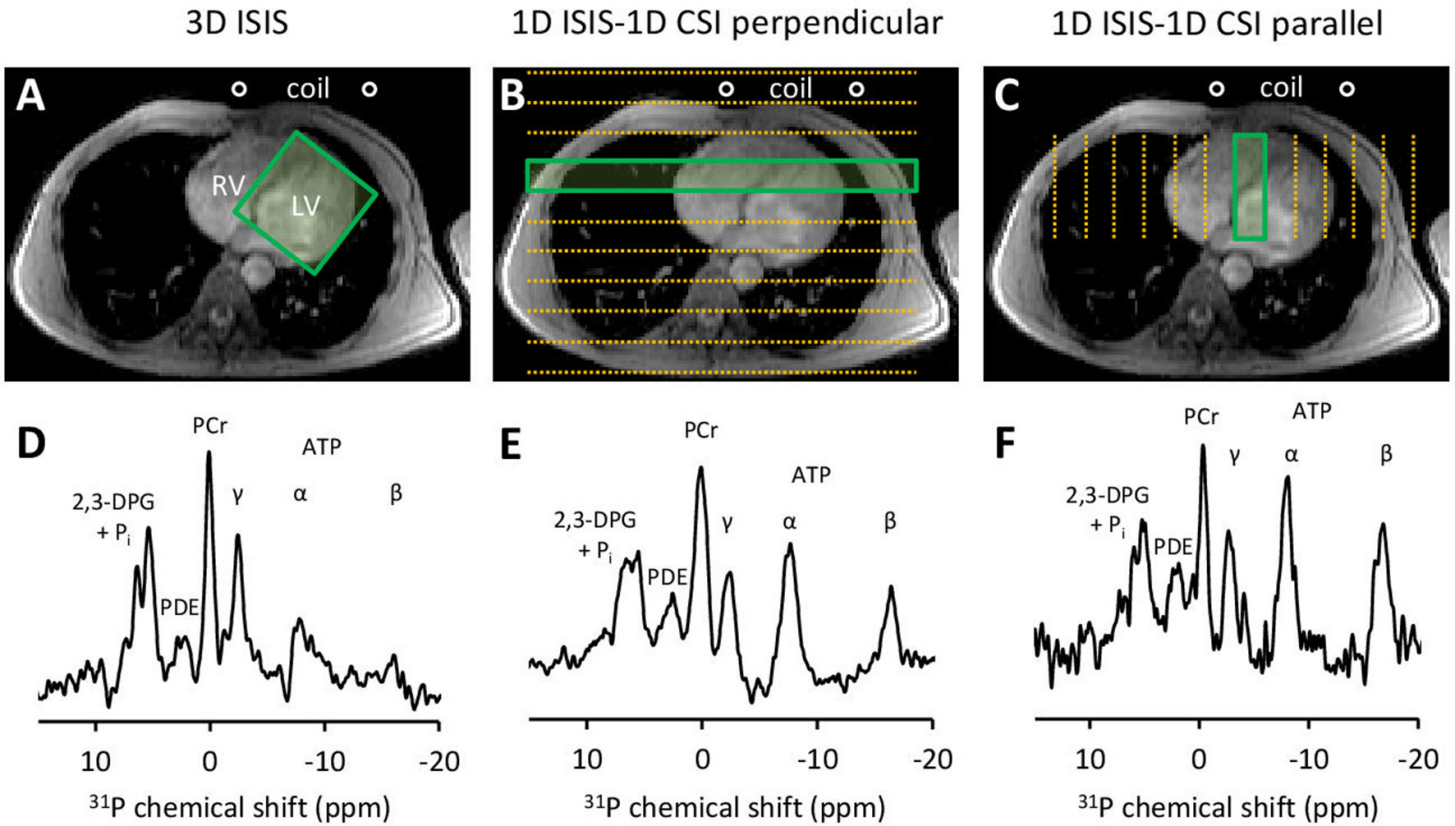
Three approaches for localized ^31^P-MRS signal acquisition of the human heart at 3 Tesla. **(A)** positioning of the 3D ISIS voxel guided by ^1^H-MRI, enclosing the whole left ventricle (LV). **(B)** 1D ISIS slice selection perpendicular to the coil with 1D CSI covering the anterior-to-posterior thorax including the LV. **(C)** 1D ISIS slice selection parallel to the coil with 1D CSI covering the left-to-right thorax including the LV. Placement of the ^31^P MR surface coil is indicated. Acquisition time per scan was ~7 min to obtain a localized ^31^P MR spectrum **(D–F)** from voxels outlined by the green boxes. 2,3-DPG, 2,3-diphosphoglycerate; ATP, adenosine triphosphate; CSI, chemical shift imaging; ISIS, image-selected *in vivo* spectroscopy; LV, left ventricle; PCr, phosphocreatine; PDE, phosphodiesters; P_i_, inorganic phosphate; RV, right ventricle.

Next, we employed three approaches (Figures 2A–C) for cardiac-triggered localized ^31^P-MRS data acquisition based on reports in the literature on obtaining non-invasive assessments of human myocardial high-energy phosphate metabolism: (1) single-voxel 3D ISIS (image-selected *in vivo* spectroscopy) (Lamb et al., 1996; Buchthal et al., 2000; Fragasso et al., 2006) requiring eight separate signal acquisitions per localization cycle, 80 × 80 × 80 mm^3^ voxel enclosing the left ventricle (LV), TR 6 ECG R-R intervals, 64 acquisitions/eight cycles; (2) 1D ISIS slice selection perpendicular to the surface coil with multi-voxel 1D CSI (chemical shift imaging) (Weiss et al., 1990; Schaefer et al., 1992) covering the anterior-to-posterior thorax including the LV, 12 phase-encoding steps, step-size 20 mm (CSI), 80 mm slice thickness (ISIS), TR 2 ECG R-R intervals, 16 averages/step; and (3) 1D ISIS slice selection parallel to the surface coil with 1D CSI covering the left-to-right thorax including the LV, 12 phase-encoding steps, step-size 20 mm (CSI), 80 mm slice thickness (ISIS), TR 2 ECG R-R intervals, 16 averages/step. Acquisition time was kept similar for all methods and was ~7 min dependent on heart rate. All procedures were performed twice to allow for assessments of method repeatability.

### *In vivo* ^31^P-MRS of human skeletal muscle

From a cohort of eight volunteers, we obtained resting-state ^31^P-MR spectra of the calf muscle to benchmark the repeatability of localized cardiac ^31^P-MRS methodology against a well-established and robust method for *in vivo* assessments of skeletal muscle energy metabolism with ^31^P-MRS (Kemp et al., 2007). Subjects were positioned supine with the ^31^P MR surface coil carefully centered underneath the calf muscle. After verifying correct positioning of the coil on ^1^H-MR scout images, a fully relaxed ^31^P-MR spectrum was acquired with adiabatic excitation, 2,048 acquisition points, and a bandwidth of 58 ppm. The procedure was repeated for an assessment of method repeatability.

### ^31^P-MRS data analysis

All spectra were processed and analyzed in jMRUI, and signal amplitudes were quantified using the AMARES time-domain fitting algorithm (Vanhamme et al., 1997) as described previously (Bakermans et al., 2015). In brief, the PCr signal was modeled by a single Lorentzian line shape at 0.00 ppm chemical shift reference. Signals of γ-ATP (doublet at −2.48 ppm), α-ATP (doublet at −7.52 ppm), and β-ATP (triplet at −16.26 ppm) were fitted to Lorentzian line shapes, equal line widths and a *J*-coupling constant of 17 Hz. A mono-exponential function was fitted to the mean saturation recovery curves of PCr, γ-ATP, α-ATP, and β-ATP to estimate the corresponding longitudinal T_1_ relaxation time constants. The *in vivo* myocardial energy status was expressed as the ratio of the PCr and γ-ATP signal amplitudes, corrected for partial saturation. Calf muscle PCr/γ-ATP ratios were quantified after fitting of the fully relaxed ^31^P-MR spectra.

### Computational modeling of myocardial energy metabolism

**Figure 3:**
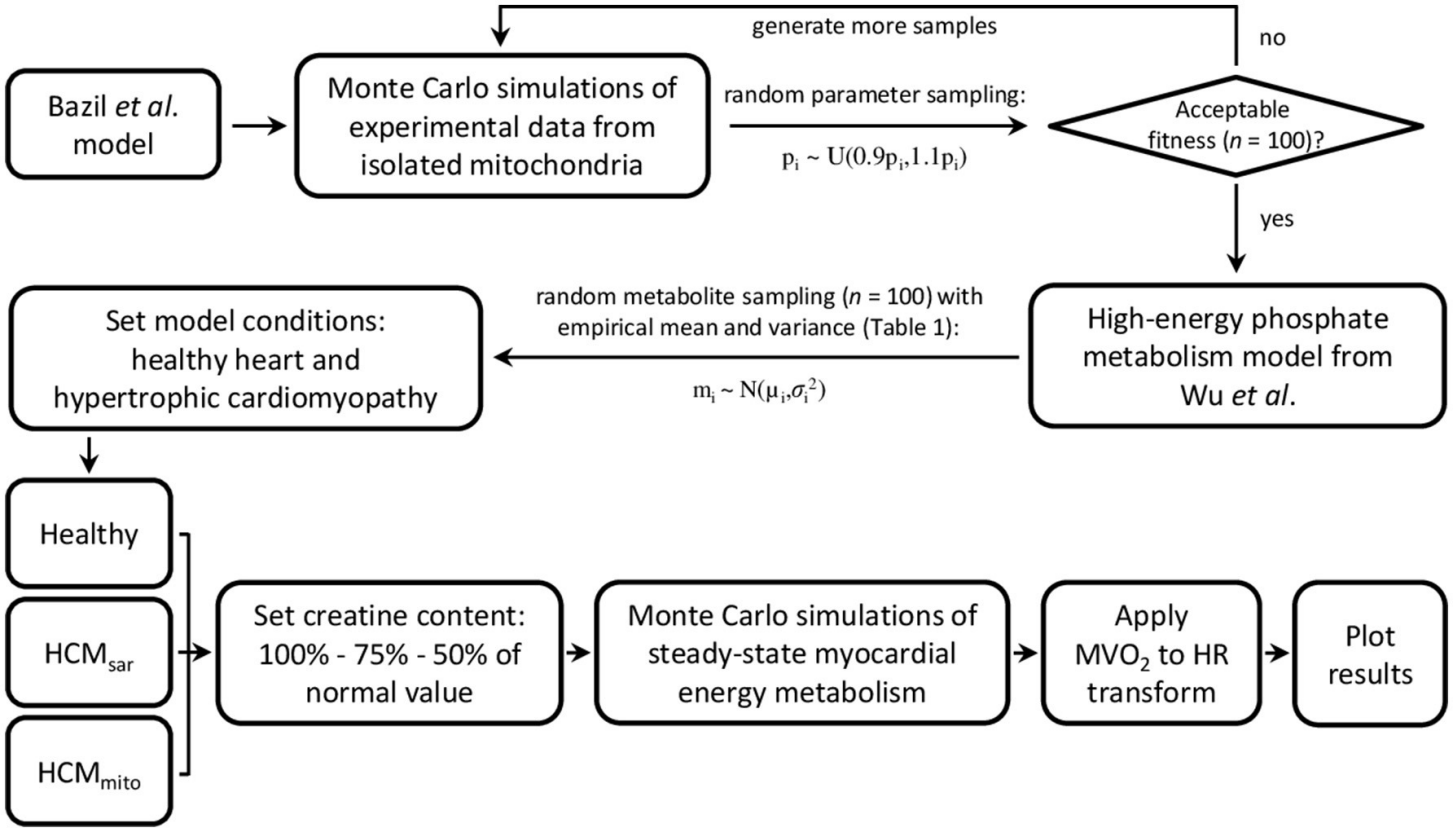
Flowchart of model parameterization and conditioning for simulations of myocardial oxidative metabolism at multiple steady-states of cardiac work rates. Bazil et al. and Wu et al. model specifics and validations are described elsewhere (Wu et al., 2008; Bazil et al., 2016). HCM, hypertrophic cardiomyopathy; HR, heart rate; MVO_2_, myocardial oxygen consumption.

Numerical simulations of myocardial PCr, ATP, ADP, and inorganic phosphate (P_i_) concentration dynamics and the resulting Gibbs free energy available from ATP hydrolysis (ΔG_P_) in response to increased cardiac work rates were performed using a biophysical model of myocardial oxidative metabolism (Figure 3). In brief, the model from Bazil et al. (2016) was supplemented with the high-energy phosphate metabolism module from Wu et al. (2008) to simulate the relationship between myocardial oxygen consumption and energy metabolism in the steady-state for healthy hearts. Mitochondrial oxygen consumption was converted to myocardial oxygen consumption (MVO_2_ in μmol min^−1^ g^−1^ LV tissue) using 5.27 g LV tissue mL^−1^ mitochondria (Vinnakota and Bassingthwaighte, 2004). The relationship between the heart rate (HR) and MVO_2_ was defined using a linear model derived from experimental data on normal human hearts (*n* = 8, *r* = 0.71, *P* = 0.048) reported in the literature (Vanoverschelde et al., 1993): MVO_2_ = HR × 0.023 + 0.82.

To estimate the impact of pathological changes in cardiomyocellular ATP consumption and ATP supply on myocardial energy homeostasis as a function of cardiac work rate in hypertrophic cardiomyopathy (HCM), two alternative model parameterizations were used (HCM_sar_ and HCM_mito_, respectively). For HCM_sar_, the linear relationship between HR and MVO_2_ was modified according to MVO_2_ = HR × 0.071–1.72 (*n* = 54, *r* = 0.79, *P* < 0.0001) (Gobel et al., 1978) to model HCM due to inefficient sarcomere ATP utilization (Ashrafian et al., 2003). Alternatively, for HCM_mito_ the mitochondrial capacity to synthesize ATP was reduced by 50% compared to healthy myocardium (Brown et al., 2016). For both models, we also explored the effect of reduced myocardial creatine content that has been documented in human HCM (Cowan, 1934; Herrmann and Decherd, 1939; Nakae et al., 2003). Hereto, additional simulations were run with reductions of the myocardial creatine pool size to 75% and to 50% of the normal value (i.e., 25% and 50% depletion, respectively). Except for the pathological adaptations of sarcomere ATP utilization, mitochondrial capacity, and creatine content as described above, the HCM model parameterizations were identical to the model parameterizations of normal myocardial oxidative metabolism.

All models were conditioned using the empirical mean myocardial PCr/ATP ratios and standard deviation (*SD*) error margins obtained with the three approaches for localized ^31^P-MRS data acquisition (Table 1). For comparison, the coefficient of variation for measurements in stationary calf muscle was used to explore the uncertainty that may be achieved by a more robust method of ^31^P-MRS data acquisition. First, Monte Carlo simulations were performed to gather model uncertainty by generating 100 random samples from a uniform distribution centered on the previously published model parameters (Bazil et al., 2016) with a range of ± 10% of their nominal value, and keeping those parameter sets that yielded deviations of model fitness within a 50% range of the least-squares error comparing model simulations with the original cardiac and calf muscle ^31^P-MRS data. Second, a sampling scheme was used to calculate the initial conditions for model metabolite concentrations, using a total creatine concentration of 41.7 ± 7.35 mM, a cytosolic ATP concentration of 8.76 ± 1.57 mM (Bottomley, 2007), and the empirical PCr/ATP ratios (Table 1). We assumed normally distributed data characterized by their means and *SD*s as reported. Initial concentrations of cytosolic ADP, adenosine monophosphate (AMP), and P_i_ were set to near zero. Both sampling schemes were used to generate the parameter sets and metabolite concentrations that were then used as inputs to drive the model and simulate the effects of work on myocardial energy variables. The mean and *SD* of the model outputs (i.e., PCr/ATP, cytosolic ADP, ΔG_P_, and P_i_) were then computed for steady-state conditions over the full physiological range of cardiac work rates (60–180 beats min^−1^). A flowchart of model parameterization and conditioning is provided in Figure 3.

**Table 1:** Results of localized ^31^P-MRS measurements of the human *in vivo* myocardial energy status at 3 Tesla.

	**3D ISIS**	**1D ISIS-1D CSI perpendicular**	**1D ISIS-1D CSI parallel**
Mean PCr/γ-ATP ± *SD* (–)	1.57 ± 0.17	2.78 ± 0.50	1.70 ± 0.56
Coefficient of variation (%)	10.8	18.0	32.9
Repeatability coefficient (–)	0.64	1.18	0.74
Repeatability coefficient (%)	40.4	42.5	43.7
Mean difference ± *SD* (–)	0.09 ± 0.33	−0.18 ± 0.61	0.11 ± 0.39
Acquisition time^*^ (s)	384	384	384

*ATP, adenosine triphosphate; CSI, chemical shift imaging; ISIS, image-selected in vivo spectroscopy; PCr, phosphocreatine*.

* *At a heart rate of 60 beats min^−1^*.

All computations were performed using MATLAB R2016a (MathWorks, Natick, MA, USA) on a Dell Precision T5810 workstation with an Intel Xeon CPU E5-2640 v3 at 2.6 GHz and 32 GB of RAM. The stiff ordinary differential equation solver ODE15s was used to simulate the model out to steady-state at each cardiac work rate.

### Statistical analyses

Data are presented as the mean ± *SD*. The coefficient of variation was defined as the ratio of the measurement *SD* to the mean, and expressed as a percentage of the mean. Repeatability of the ^31^P-MRS methods was assessed using Bland-Altman analyses of the PCr/γ-ATP ratios (Bland and Altman, 1986). The repeatability coefficient is given by 1.96 times the *SD* of the difference between the two consecutive measurements, and was expressed as a percentage of the mean PCr/γ-ATP ratio.

## Results

We acquired ^31^P-MR spectra of the human heart using three approaches for localized signal acquisition (Figure 2). All spectra feature the distinct resonance peak of the high-energy phosphate PCr (chemical shift reference at 0.00 ppm). The three phosphate groups in ATP (α-, β-, and γ-ATP) are reflected by three multiplets at different chemical shifts upfield of PCr. Phosphodiesters (PDE) give rise to the peak at 3 ppm. Two peaks associated with 2,3-diphosphoglycerate (2,3-DPG) in erythrocytes in the ventricular blood appear further downfield of PCr. These peaks overlap with P_i_ resonating at a pH-dependent chemical shift of ~5 ppm relative to PCr. Contamination of the spectra with signal from 2,3-DPG in the blood prevented estimations of myocardial pH using the P_i_-PCr chemical shift difference. Non-localized saturation recovery experiments of the chest yielded T_1_ relaxation time constants for high-energy phosphate metabolites at 3 Tesla, and were 4.9 s for PCr, 1.9 s for γ-ATP, 2.7 s for α-ATP, and 3.1 s for β-ATP. These values were used to correct the observed PCr/γ-ATP ratios for partial saturation effects at the heart rate-dependent TR of localized ^31^P-MRS acquisitions.

### Localized ^31^P-MRS measurement repeatability of the *in vivo* PCr/ATP ratio

**Figure 4:**
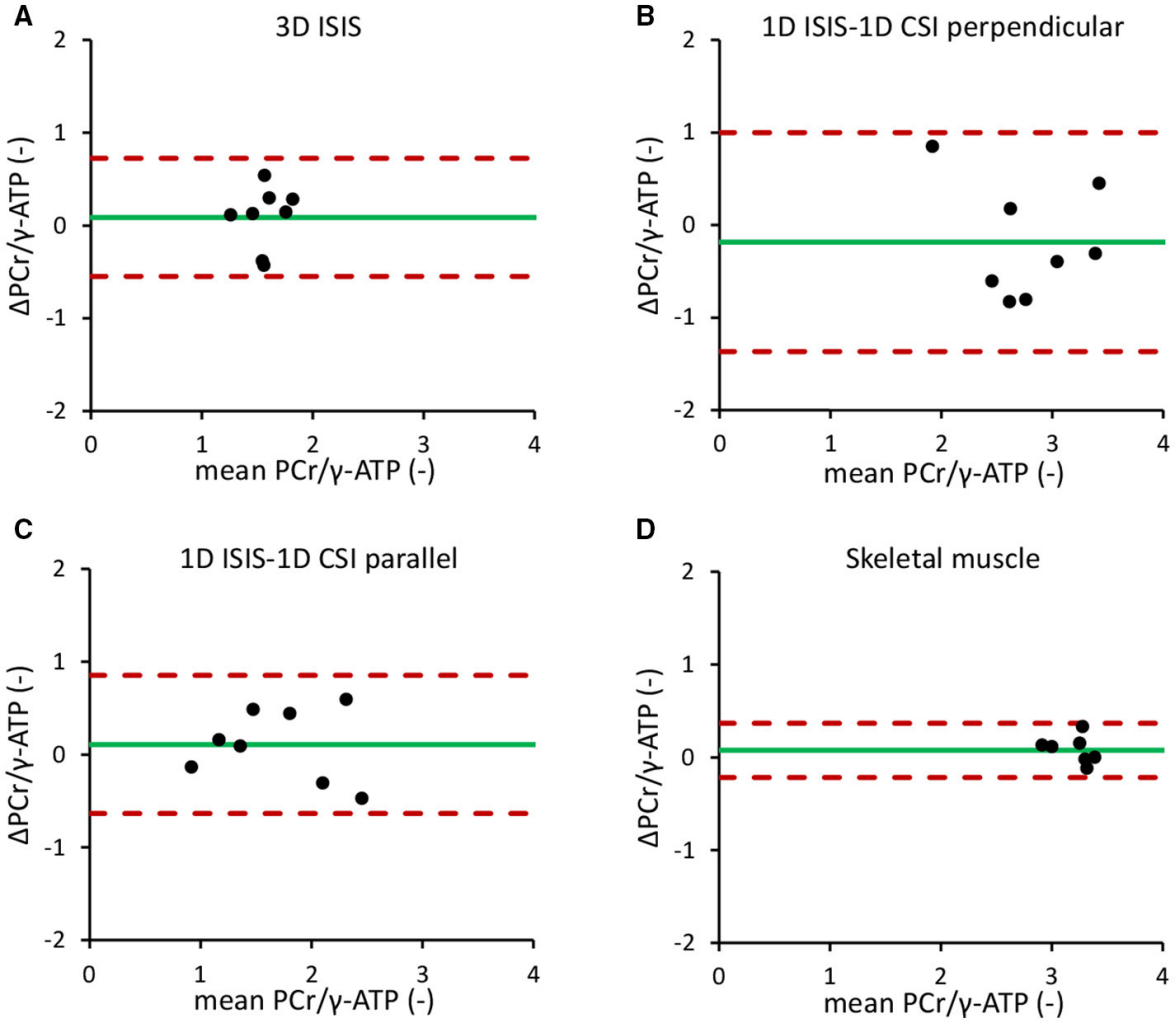
Bland-Altman repeatability analyses of localized ^31^P-MRS measurements of the *in vivo* myocardial PCr/γ-ATP ratio. **(A)** 3D ISIS of the human heart. **(B)** 1D CSI in a 1D ISIS-selected slice perpendicular to the surface coil. **(C)** 1D CSI in a 1D ISIS-selected slice parallel to the surface coil. **(D)** ^31^P-MRS of the human calf muscle. Dotted red lines indicate the 1.96 × *SD* limits of the difference between two measurements in the same subject (repeatability coefficient) around the mean difference (green solid line). ATP, adenosine triphosphate; CSI, chemical shift imaging; ISIS, image-selected *in vivo* spectroscopy; PCr, phosphocreatine.

The mean *in vivo* myocardial PCr/γ-ATP ratio measured with single-voxel localized 3D ISIS in normal volunteers (*n* = 8) was 1.57 ± 0.17 with a mean difference between measurements of 0.09 ± 0.33 and a repeatability coefficient of 40.4%. For multi-voxel 1D CSI in a 1D ISIS-selected slice perpendicular to the surface coil, the PCr/γ-ATP ratio was 2.78 ± 0.50 with a mean difference of −0.18 ± 0.61 and a repeatability coefficient of 42.5%. Alternatively, with 1D CSI in a 1D ISIS-selected slice parallel to the surface coil, the PCr/γ-ATP ratio was 1.70 ± 0.56 with a mean difference of 0.11 ± 0.39 and a repeatability coefficient of 43.7%. The results of these Bland-Altman analyses are displayed in Figures 4A–C and summarized in Table 1.

The repeatability coefficient for ^31^P-MRS measurements of the *in vivo* calf muscle PCr/γ-ATP ratio was only 9.0%. Calf muscle PCr/γ-ATP was 3.25 ± 0.21 with a mean difference between measurements of 0.08 ± 0.13 (Figure 4D).

### Model predictions of myocardial energy homeostasis during exercise

**Figure 5:**
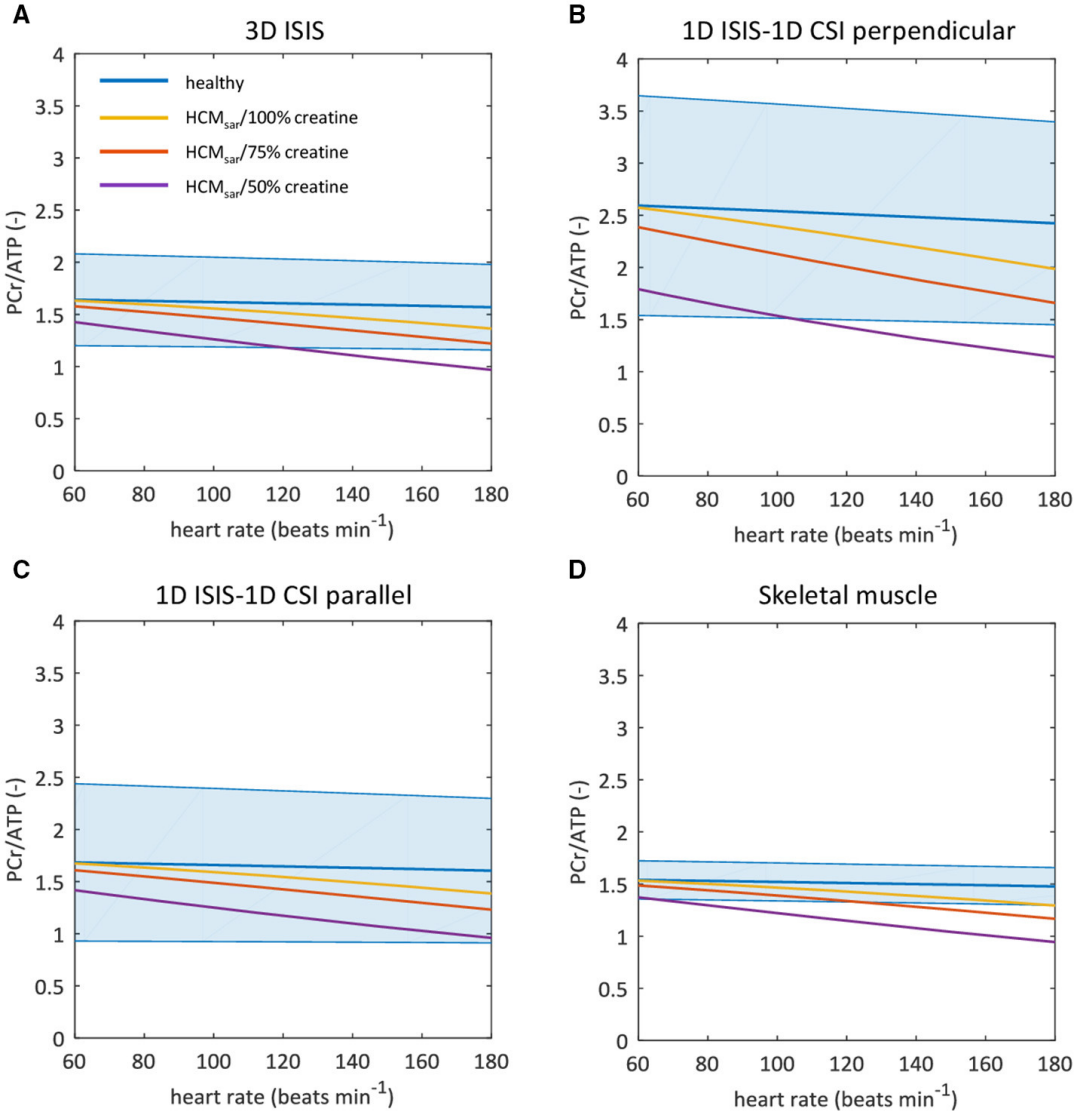
Sarcomeric energy inefficiency results in lower myocardial PCr/ATP ratios compared to the healthy myocardium. Graphs show computational model-based predictions of the myocardial PCr/ATP ratio over a range of cardiac work rates for the healthy heart (blue) and for hypertrophic cardiomyopathy due to sarcomeric energy inefficiency (HCM_sar_) with 100% (yellow), 75% (red), and 50% (purple) of the normal creatine pool size. The solid curve represents the mean of the simulation results while the shaded region reflects the model uncertainty (95% confidence intervals). Resting-state mean and confidence intervals are based on empirical ^31^P-MRS measurements and associated coefficients of variation for 3D ISIS **(A)**, 1D CSI in a 1D ISIS-selected slice perpendicular to the surface coil **(B)**, 1D CSI in a 1D ISIS-selected slice parallel to the surface coil **(C)**, and for the improved measurement precision of ^31^P-MRS in calf skeletal muscle but assuming the mean PCr/ATP ratio found with 3D ISIS **(D)**. ATP, adenosine triphosphate; CSI, chemical shift imaging; ISIS, image-selected *in vivo* spectroscopy; PCr, phosphocreatine.

The empirical resting-state ^31^P-MR data obtained with each of the three approaches for localized signal acquisition were used to condition the model. The resulting model predictions of the myocardial PCr/ATP ratio for higher cardiac work rates are plotted with 95% confidence intervals in Figures 5A–C. To benchmark the current practice against measurements with higher precision, Figure 5D shows the model predictions initiated with the mean myocardial PCr/ATP ratio measured with 3D ISIS (1.57), but with the smaller coefficient of variation that was obtained for ^31^P-MRS of calf muscle (6.4%). The model predicted an approximate maximal reduction of only 10% of the steady-state myocardial PCr/ATP ratio over the entire physiological range of cardiac work rates in normal human hearts (blue curves in Figure 5). Inefficient sarcomere energy utilization as modeled by HCM_sar_ led to a markedly decreased PCr/ATP ratio for high cardiac work rates (yellow curves in Figure 5), which was exacerbated by depletion of the myocardial creatine pool to 75% (red curves) and to 50% (purple curves) of the normal value.

**Figure 6:**
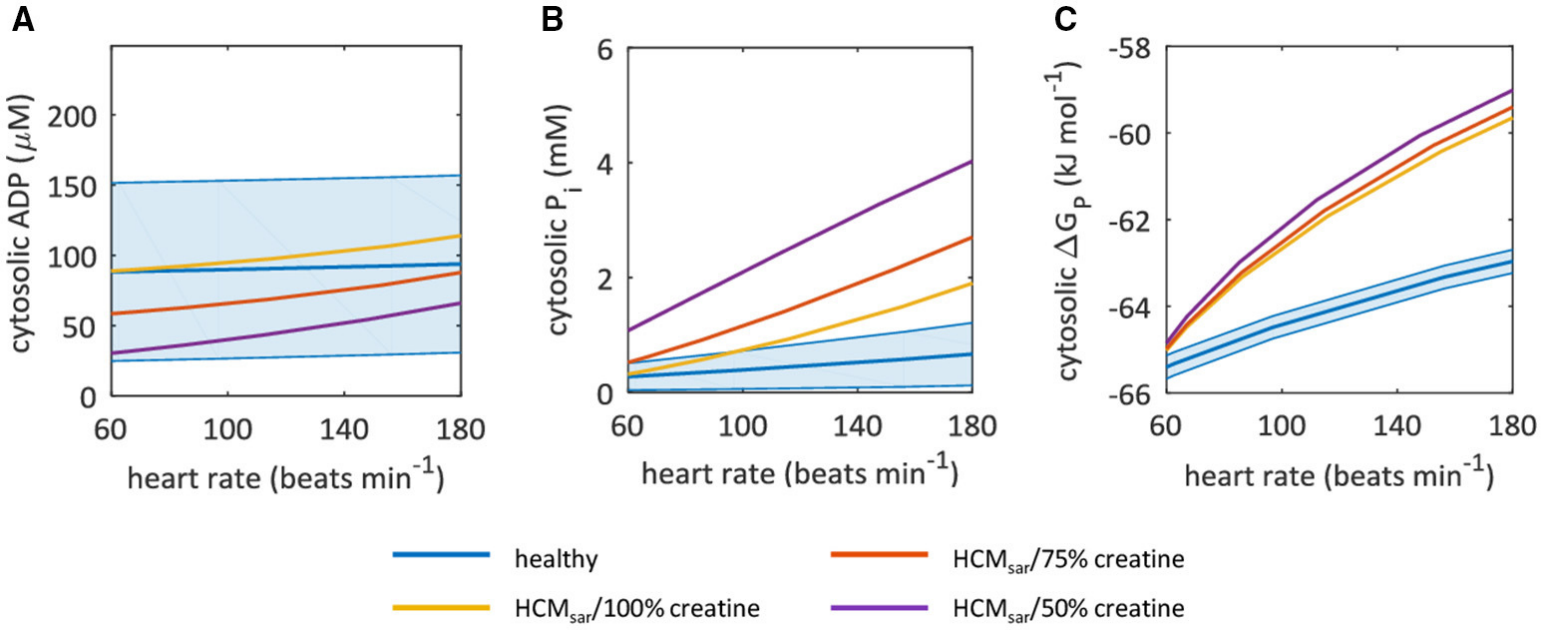
Sarcomeric energy inefficiency leads to an increase in myocardial cytosolic P_i_ concentration for high cardiac work rates. Graphs show computational model-based predictions of myocardial energy homeostasis over a range of cardiac work rates for the healthy heart (blue) and for hypertrophic cardiomyopathy due to sarcomeric energy inefficiency (HCM_sar_) with 100% (yellow), 75% (red), and 50% (purple) of the normal creatine pool size. The solid curve represents the mean of the simulation results while the shaded region reflects the model uncertainty (95% confidence intervals). Resting-state mean and confidence intervals are based on empirical ^31^P-MRS measurements and associated coefficient of variation for 3D ISIS. **(A)** cytosolic ADP concentrations. **(B)** cytosolic P_i_ concentrations. **(C)** Gibbs free energy available from ATP hydrolysis ΔG_P_. ADP, adenosine diphosphate; ISIS, image-selected *in vivo* spectroscopy; P_i_, inorganic phosphate.

Other model outputs on myocardial energy homeostasis are plotted in Figure 6, using the *in vivo* myocardial PCr/ATP measured with 3D ISIS localization (Table 1) to condition the model. Cytosolic ADP (Figure 6A) and P_i_ (Figure 6B) remained nearly constant in the normal myocardium over the entire range of cardiac work rates, whereas the Gibbs free energy available from ATP hydrolysis ΔG_P_ decreased in magnitude by ~2 kJ mol^−1^ for heart rates up to 180 beats min^−1^ (Figure 6C). The value of ΔG_P_ calculated here differed from previous reports for normal hearts at rest (Weiss et al., 2005), because we used a different more accurate estimate of the reference Gibbs free energy ΔG^0^ (Li et al., 2011) than adopted in prior studies (Weiss et al., 2005).

When sarcomere energy utilization is inefficient, the HCM_sar_ model predicted a similar ΔG_P_ at rest but a 2.5-fold larger decrease in magnitude of ΔG_P_ for heart rates of 180 beats min^−1^ compared with the normal myocardium (yellow curve in Figure 6C). This result was similar for simulations with reduced myocardial creatine pool sizes (75 and 50% of the normal creatine level; red and purple curves in Figure 6C, respectively), and can mainly be attributed to the steeper changes in myocardial P_i_ concentrations in response to exercise than in the normal myocardium: P_i_ concentration increased almost 3-fold compared to a less than 100% increase in ADP concentration for HCM_sar_ with 50% of the normal creatine level (Figures 6A,B). The predicted myocardial ADP concentrations fell with more severe myocardial creatine depletion (Figure 6A).

**Figure 7:**
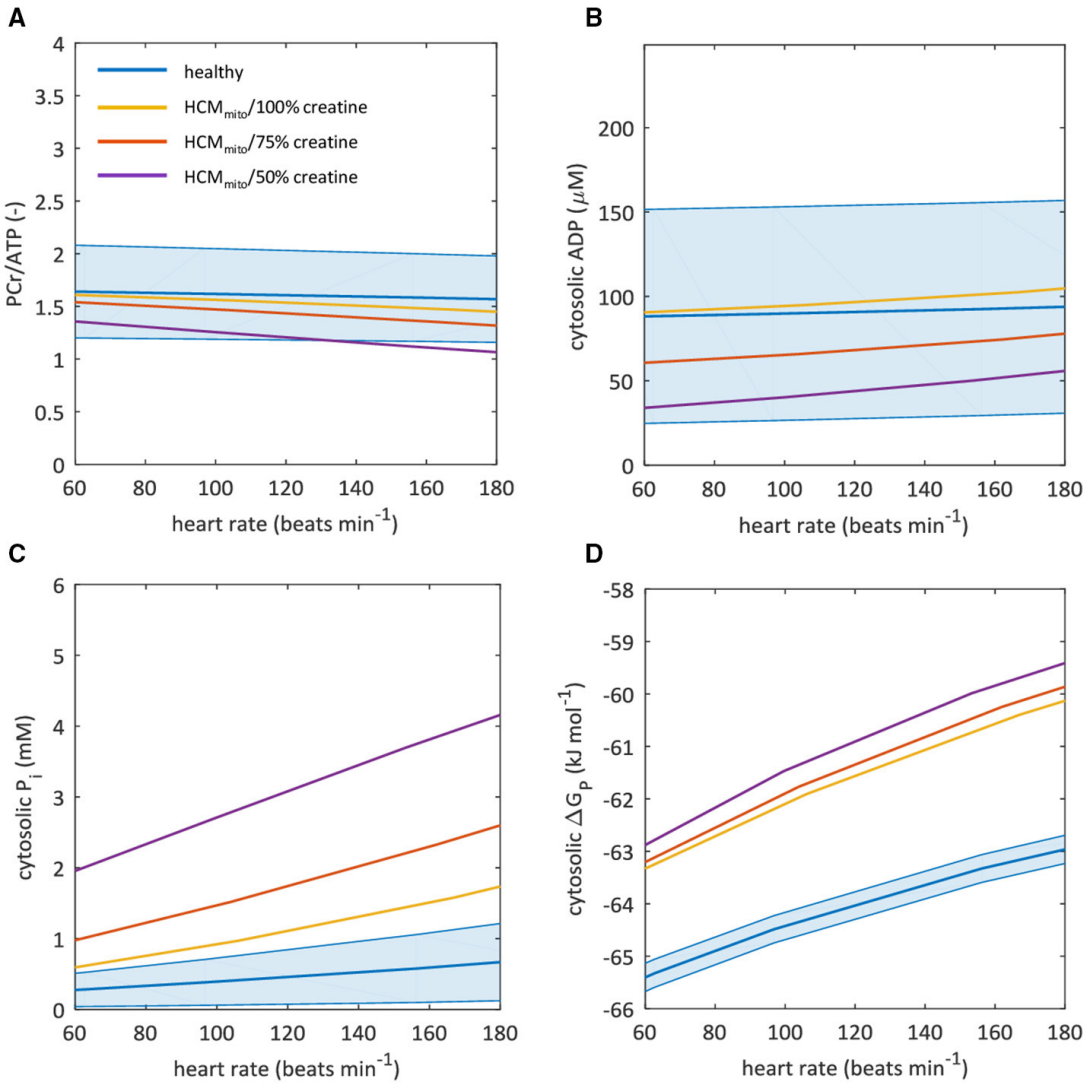
A reduction in mitochondrial capacity to synthesize ATP disturbs myocardial energy homeostasis. Graphs show computational model-based predictions of myocardial energy homeostasis over a range of cardiac work rates for the healthy heart (blue) and for hypertrophic cardiomyopathy due to a 50% reduction in mitochondrial capacity to synthesize ATP (HCM_mito_) with 100% (yellow), 75% (red), and 50% (purple) of the normal creatine pool size. The solid curve represents the mean of the simulation results while the shaded region reflects the model uncertainty (95% confidence intervals). Resting-state mean and confidence intervals are based on empirical ^31^P-MRS measurements and associated coefficient of variation for 3D ISIS. **(A)** myocardial PCr/ATP ratio. **(B)** cytosolic ADP concentrations. **(C)** cytosolic P_i_ concentrations. **(D)** Gibbs free energy available from ATP hydrolysis ΔG_P_. ADP, adenosine diphosphate; ATP, adenosine triphosphate; ISIS, image-selected *in vivo* spectroscopy; PCr, phosphocreatine; P_i_, inorganic phosphate.

Simulations of a reduced mitochondrial capacity to produce ATP (HCM_mito_) for steady-state conditions over a range of cardiac work rates are compared with normal myocardial energy homeostasis in Figures 7A–D. The predicted decrease in magnitude of ΔG_P_ for increased cardiac work rates was similar to the normal myocardium (yellow curve in Figure 7D). However, the absolute Gibbs free energy of ATP hydrolysis was nearly 2.3 kJ mol^−1^ lower over the full physiological range of cardiac work rates compared to normal myocardium. Consequently, the predicted minimal magnitude of ΔG_P_ attained during increased cardiac work rates in conditions of reduced mitochondrial capacity was similar to model predictions for ΔG_P_ when sarcomere energy utilization is inefficient: ~−60 kJ mol^−1^. Also, the predicted magnitude of ΔG_P_ was similar in simulations of myocardial creatine depletion. Figure 7C illustrates that this was due in large part to steeper changes in myocardial P_i_ concentrations in response to increased cardiac work rates compared to the normal myocardium for which P_i_ content remained nearly constant. Predicted myocardial ADP concentrations were lower for reduced myocardial creatine pool sizes (Figure 7B).

Taken together, these simulations illustrate that when conducting cardiac ^31^P-MRS exercise stress testing with large measurement error margins, results obtained under pathophysiologic conditions such as sarcomeric energy inefficiency, reduced mitochondrial capacity, and creatine depletion may still lie well within the 95% confidence interval of normal myocardial PCr/ATP dynamics.

## Discussion

The non-invasive nature of localized ^31^P-MRS makes this technique a candidate modality for measurements of *in vivo* human myocardial energy metabolism during cardiac stress tests. Despite this promising potential, practical and technological challenges have prevented ^31^P-MRS from becoming a widespread diagnostic imaging modality in the clinical workflow. Here, we determined the resting-state measurement variability and repeatability for three commonly used approaches for localized ^31^P-MRS of the human heart, and employed computational modeling to estimate their suitability for assessments of myocardial energy homeostasis over a broad range of cardiac work rates. Our results show that with the level of precision achieved by current methodology, altered energy homeostasis under pathophysiologic conditions such as decreased mitochondrial capacity or inefficient sarcomere energy utilization may not be detectable with cardiac ^31^P-MRS stress testing.

For the healthy human myocardium at rest, the literature mean value of the PCr/ATP ratio is ~1.7 ± 0.3 (Bottomley, 2007). However, normal myocardial PCr/ATP values reported for healthy subjects range from 0.9 ± 0.3 up to 2.5 ± 0.5, demonstrating a large variability among research sites that use different ^31^P-MRS methods for quantification of the human myocardial energy status (Figure 1, also comprehensively reviewed in Bottomley, 2007). Our results were corrected for heart rate-dependent partial saturation effects that could modulate the PCr/ATP ratio for our measurements at relatively short TR (i.e., TR < 5 × T_1_). Nonetheless, for the three localization approaches applied in the same subjects, normal PCr/ATP ratios in the present work ranged from 1.57 ± 0.17 for single-voxel 3D ISIS up to 2.78 ± 0.50 for multi-voxel 1D CSI with 1D ISIS slice selection perpendicular to the surface coil. Such differences between methods may be attributed to different degrees of signal contamination. Indeed, single-voxel 3D ISIS generally benefits from a well-defined voxel shape (de Graaf, 2008), whereas CSI may suffer from Fourier bleeding that introduces signal contamination originating from tissue in voxels outside the region of interest (Keevil, 2006). Particularly in 1D CSI oriented perpendicular to the surface coil, Fourier bleeding of signal from high PCr levels present in superficial chest skeletal muscle may contribute to an overestimation of the actual myocardial PCr/ATP ratio, compromise measurement precision, and ultimately hamper the detection of changes in myocardial PCr levels. Moreover, experimental variation in surface coil placement and voxel positioning negatively affects measurement repeatability, compromising the applicability of current ^31^P-MRS methodology for diagnostic cardiac stress testing.

Only few laboratories have reported on method repeatability (Bland and Altman, 1986) of human cardiac ^31^P-MRS in test-retest study designs. Lamb et al. compared several signal acquisition localization schemes at 1.5 Tesla (Lamb et al., 1996), and found that the inter-examination repeatability coefficient for the PCr/ATP ratio was rather large: >45% for 1D CSI, 1D CSI with 2D ISIS, as well as for 3D ISIS. This was predominantly attributed to differences in coil placement and other practicalities between examinations rather than true physiological changes in the myocardium (e.g., of nutritional origin). Using 1D CSI, Schaefer et al. reported a test-retest repeatability coefficient of 22% for measurements of the human myocardial PCr/ATP ratio at 1.5 Tesla (Schaefer et al., 1992). The use of magnetic field strengths >1.5 T holds promise in terms of improved signal to noise ratios and/or shorter acquisition times, which is theoretically beneficial for signal quantification and therewith measurement repeatability. A repeatability coefficient of 53% was reported for ^31^P-MRS measurements of the PCr/ATP ratio with 31 min of acquisition time using 3D CSI at 3 Tesla (Tyler et al., 2009). Later, this protocol was adjusted by Dass et al. to achieve a clinically acceptable acquisition time of 8 min by lowering the 3D CSI spatial resolution and omitting cardiac triggering (Dass et al., 2010), but with unreported measurement repeatability. Similar to these reports in the literature, we found rather large repeatability coefficients of more than 40% for the myocardial PCr/ATP ratios obtained within 7 min of acquisition time. Clearly, these data suggest that with the strategies currently used for cardiac ^31^P-MRS, only large changes in the PCr/ATP ratio may be detected in the human myocardium. As such, ^31^P-MRS measurement of *in vivo* myocardial PCr/ATP ratio in humans has not evolved beyond its use as a research tool to study myocardial energy homeostasis in groups of patients with phenotypic cardiomyopathy (Lamb et al., 1999; Dass et al., 2015; Levelt et al., 2016).

Our simulations showed that a 50% reduction of the mitochondrial capacity to produce ATP results in only a small decrease of the PCr/ATP ratio at increased cardiac work rates. Due to a lack of nutrients and oxygen, mitochondrial ATP production may become marginal in ischemic conditions, leading to a more pronounced decrease of the PCr/ATP ratio during exercise. Indeed, in some cases, cardiac ^31^P-MRS exercise stress testing has provided encouraging results. Particularly, a transient exercise-induced decrease in the myocardial PCr/ATP ratio was observed in patients with coronary artery disease, which could not be detected in patients with non-ischemic heart disease (Weiss et al., 1990). This response improved after successful revascularization, suggesting clinical potential for this methodology in myocardial ischemia. Similarly, cardiac ^31^P-MRS exercise stress testing has been proposed as a means to noninvasively test therapeutic strategies in Chagas disease, where a reduction in the PCr/ATP ratio may be indicative of microvascular disease caused by a *Trypanosoma cruzi* parasite infection (Betim Paes Leme et al., 2013).

Furthermore, our simulations indicate that the normal myocardial P_i_ concentration is tightly regulated and maintained within a submillimolar range over the entire physiological range of cardiac work rates. In contrast, for both models of HCM, cytosolic P_i_ was predicted to increase to millimolar concentration levels approaching 2 mM at high cardiac work rates. Indeed, a rise in P_i_ has been reported for patients with hypertensive heart disease after pharmacologically induced stress (Lamb et al., 1999). Moreover, the HCM models predicted that myocardial creatine depletion (Cowan, 1934; Herrmann and Decherd, 1939) progressively aggravates the loss of cytosolic P_i_ concentration homeostasis. Combined, our simulations support the quantitative hypothesis of cytosolic P_i_ interference with myocardial mechanical function as proposed by Tewari et al. (2016a), which may explain progressive heart failure in human HCM. Notably, the predicted rise of cytosolic P_i_ concentrations into the millimolar range for higher cardiac work rates in HCM makes this metabolite an alternative target for diagnostic *in vivo* detection with ^31^P-MRS. This will, however, require major methodological improvements in terms of signal localization to prevent any contaminating signal from 2,3-DPG in the blood overlapping with P_i_.

Finally, our model predictions showed that ^31^P-MRS measurements of the steady-state PCr/ATP ratio at high-intensity cardiac work rates are more sensitive to pathophysiological derangements in myocardial energy homeostasis than resting-state measurements. Protocols to perform such strenuous physical exercise inside a clinical MR scanner as an alternative to pharmacologically induced stressors have recently been developed (Jeneson et al., 2010; Gusso et al., 2012) and applied to study heart function (La Gerche et al., 2013) and perfusion (Pflugi et al., 2015) at heart rates > 160 beats min^−1^. These protocols utilize a supine cycling exercise regime rather than isometric hand grip exercise (Weiss et al., 1990) or prone flexion of the legs (Hudsmith et al., 2009), and therefore facilitate a broad range of cardiac work rates. However, cycling motion of the legs combined with higher respiratory and heart rates may aggravate motion artifacts and signal contamination in localized ^31^P-MRS, deteriorate the ECG signal typically used for synchronizing measurements with the beating heart, and introduce magnetic field inhomogeneities that can compromise data quality. These aspects make obtaining quantitative results with ^31^P-MRS during high-intensity exercise even more challenging than at resting-state conditions, and are obviously detrimental to measurement precision. Moreover, strenuous exercise cannot be maintained at a steady-state level for a prolonged period of time, particularly in case of myocardial ischemia or other pathophysiological conditions, which imposes practical constraints on data acquisition time. Ongoing developments in coil design for radiofrequency transmission and signal reception (El-Sharkawy et al., 2009; Rodgers and Robson, 2015; Schaller et al., 2015; Löring et al., 2016), MR sequence design (Robson et al., 2005), and subsequent data processing (Zhang et al., 2013) for ^31^P-MRS may alleviate these issues by increasing sensitivity, spatial localization, and decreasing acquisition time. In addition, clinical MR scanners with a magnetic field strength of 7 Tesla are becoming more widely available and promise higher signal to noise ratios and a potential for higher spectral resolution (Stoll et al., 2016). Indeed, spectra with a signal to noise ratio similar to those acquired in 30 min at 3 Tesla were acquired in only 6 min at 7 Tesla, aided by the shorter longitudinal T_1_ relaxation times for high-energy phosphate metabolites at 7 Tesla (Rodgers et al., 2014). On the other hand, higher magnetic field strengths require even more demanding solutions for minimizing magnetic field inhomogeneities and motion-induced artifacts that could diminish the theoretical gain in sensitivity and/or spectral, spatial, and temporal resolution.

The current work emphasizes the need for technological and methodological advancements of cardiac ^31^P-MRS. In addition, improvements are required for an experimental validation of computational model predictions of human myocardial energy homeostasis and its (mal-)adaptation in disease. Currently, such validations have been limited to *in vitro* assays and *in vivo* studies with animal models (Wu et al., 2008, 2009). Further developments in ^31^P-MRS methodology may lead to opportunities for *in vivo* model validation, and ultimately for cardiac ^31^P-MRS exercise stress testing to become of any diagnostic merit.

## Conclusion

Simulations of human myocardial energy homeostasis over a broad range of cardiac work rates predict only moderate changes in the PCr/ATP ratio, even for hypertrophic cardiomyopathy at high-intensity work rates. The present study shows that current measurement precision of commonly used localized ^31^P-MRS methods for quantification of the myocardial PCr/ATP ratio precludes the detection of such changes. This prevents using ^31^P-MRS for diagnostic testing and risk stratification in the clinic. As such, these results warrant further developments in ^31^P-MRS methodology combined with more strenuous exercise stress testing protocols to facilitate *in vivo* cardiac ^31^P-MRS exercise stress testing of myocardial energy metabolism in patients.

## Author contributions

AB, JB, DB, and JJ contributed to the design of the study. AB acquired and analyzed the cardiac ^31^P-MRS data. JB provided the computational model simulations. All authors contributed to the interpretation of the data. AB, JB, and JJ drafted the manuscript. AN, GS, SB, DB, and JJ critically revised the work for important intellectual content. All authors approved the final version of the manuscript, and agree to be accountable for the content of the work.

### Conflict of interest statement

The authors declare that the research was conducted in the absence of any commercial or financial relationships that could be construed as a potential conflict of interest. The handling Editor declared a past co-authorship with the authors GS and AN.

## Abbreviations

2,3-DPG: 2,3-diphosphoglycerate
^31^P-MRS: phosphorus-31 magnetic resonance spectroscopy
ADP: adenosine diphosphate
AMP: adenosine monophosphate
ATP: adenosine triphosphate
CSI: chemical shift imaging
ECG: electrocardiogram
HCM: hypertrophic cardiomyopathy
HR: heart rate
ISIS: image-selected *in vivo* spectroscopy
LV: left ventricle
MRI: magnetic resonance imaging
MVO_2_: myocardial oxygen consumption
PCr: phosphocreatine
PDE: phosphodiesters
P_i_: inorganic phosphate
RV: right ventricle
SD: standard deviation
TR: repetition time.
